# Full-endoscopic transpedicular discectomy (FETD) for lumbar herniations: Case report and review of the literature

**DOI:** 10.1016/j.ijscr.2020.05.085

**Published:** 2020-06-09

**Authors:** Enrico Giordan, Jacopo Del Verme, Flaminia Coluzzi, Giuseppe Canova, Domenico Billeci

**Affiliations:** a Department of Neurosurgery, Aulss 2 Marca Trevigiana, Treviso, Veneto, Italy; b Department of Medical and Surgical Sciences and Biotechnologies, Sapienza University of Rome, Polo Pontino, Latina, Italy

**Keywords:** Transpedicular, Endoscopic, Discectomy, Mini-invasive, Percutaneous

## Abstract

**Introduction:**

One of the most challenging occurrences in full-endoscopic surgery for lumbar disc protrusions are up-migrated or down-migrated herniations. Those occurrences are difficult to retrieve with transforaminal or interlaminar approaches.

**Presentation of case:**

We describe our experience in dealing with a right paramedian down-migrated L3-L4 disc herniation. The patient underwent full endoscopic transpedicular endoscopic discectomy (FETD), by reaming the right L4 peduncle for intracanal access and fragment retrieval. We also reviewed the recent literature to summarize the advantages of transpedicular approaches, along with current indications and contraindications for this procedure.

**Discussion:**

We highlighted how FETD is safe and feasible for down-migrated and up-migrated disc herniation showing excellent results in our patient and in the small cohorts of patients already published in the literature.

**Conclusion:**

FETD was effective in treating up-migrated and down-migrated disc herniation, as well as discal cysts, showing the feasibility and safety of the technique from any level from L1 to S1.

## Highlights

• The full-endoscopic transpedicular approach could be useful in treating lumbar migrated disc herniations or juxtafacet cysts.
• No documented risk of pedicle fracture after FETD.
• FETD showed feasibility and safety from any level from L1 to S1.
• Potentially FETD may be used for drainage of epidural abscesses and epidural hematomas, or in case intra-canal biopsies are needed.

## 1 Introduction

Full-endoscopic spine surgery takes advantage of the high-definition magnification positioned proximally to the target pathology. Despite technological improvements, the rising number of studies describing the safety and feasibility of full endoscopic procedures, and the documented advantages over traditional open discectomies [1,2], some authors still believe that endoscopic discectomy should be limited to very selected cases, tailoring the indications on fragment location or patient anatomy [3].

Some of the most challenging conditions are up-migrated or down-migrated lumbar disk herniations. Here we describe a paramedian down-migrated L3-L4 disc herniation. The patient underwent full-endoscopic transpedicular discectomy (FETD), by reaming the right L4 pedicle for intracanal access. We also reviewed the recent literature to summarize the advantages, along with indications and contraindications, of transpedicular approaches. This work has been reported in line with the SCARE criteria [4].

## 2 Case presentation and surgical technique

**Fig. 1:**
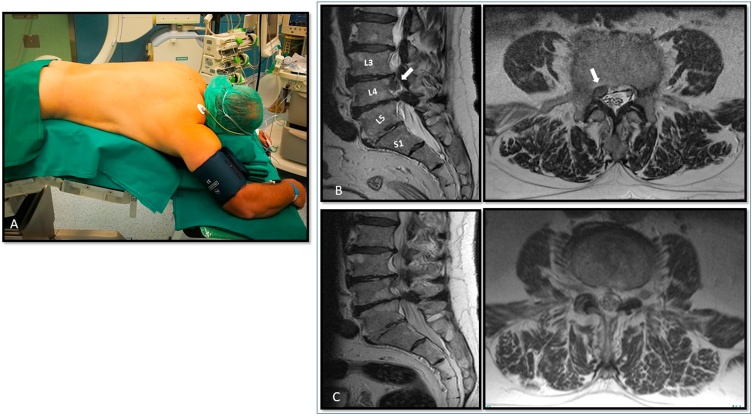
**A**. patient positioned on a prone position on the operating table. The patient is awake, only mildly sedated, waiting for the local anesthesia and the procedure to start. **B**. Preoperative sagittal (*left*) and axial (*right*) T2-weighted lumbar MRI scan showing a right L3-L4 paramedian disc herniation caudally migrated. **C**. Three-months postoperative T2-weighted lumbar MRI scan shows the satisfactory outcome of the procedure and the lack of signal intensity suggestive for pedicle damage or fracture.

A 76-year-old man presented with a 12-month history of low back pain radiating down his right leg, without sensory dysfunction or motor weakness. Preoperative pain intensity was 8 on a 10-point scale and unresponsive to medication (i.e., continuous oral NSAIDs and 6 weeks of opioids). Preoperative MRI showed a paramedian, down-migrated, L3-L4 disc herniation **(**Fig. 1**, B)**. Given the concordance between clinical symptoms and MRI findings, a right L3-L4 FETD was planned. From a prone position (Fig. 1**, A)**, the patient underwent the procedure under monitored and local anesthesia (local infiltration with a 50/50 mixture of lidocaine 2% and physiological solution). Preoperatively midazolam 2 mg was intravenously administered for anxiety control. Propofol was administrated by continuous target-controlled infusion to achieve conscious sedation with spontaneous ventilation [5].

**Fig. 2:**
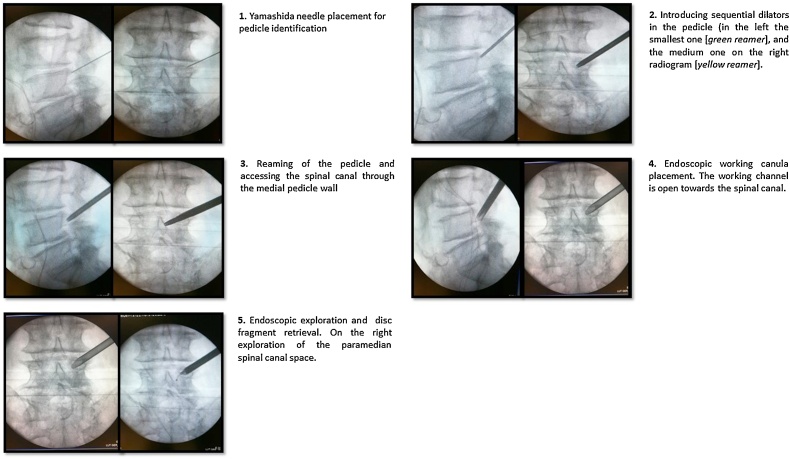
Form upper left to lower left on clockwork disposition, and from number 1 to 5, AP (*left*) and lateral (*right*) radiograms of sequential steps of the transpedicular discectomy procedure. Descriptions on the right side.

**Fig. 3:**
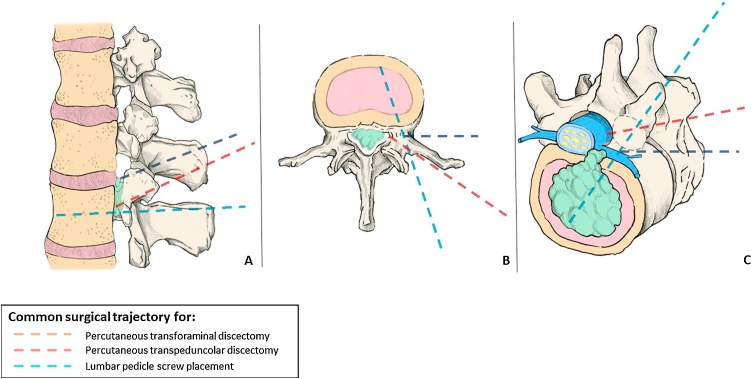
Sagittal (**A**), axial (**B**), and oblique 3-dimensional (**C**) pictorial representation of different access routes for spinal surgery procedures. Comparison between endoscopic transforaminal, endoscopic transpedicular, and pedicle screw surgery.

The Joimax TESSYS*®* (*Joimax®* GmbH, Karlsruhe, Germany) transforaminal endoscopic surgical system was used. We established the paramedian skin entry point, under fluoroscopic guidance, at approximately 10−11 cm lateral to the L4 spinous process. Using intermittent fluoroscopic guidance, alternating between lateral and anterior-posterior views, an 18-gauge needle was advanced until reaching the lateral border of the right L4 peduncle (Fig. 2,1). Then it was replaced by a soft Kirshner wire that served as a guide for introducing sequential dilators. The smallest dilatator (green guiding rod, outer diameter: 1.8 mm) was hammered inside the right L4 pedicle (Fig. 2,2). The trajectory was forced towards the medial wall of the pedicle to access the intracanal space (Fig. 3) After radiographic confirmation of the trajectory, the hole in the pedicle was increased with subsequential dilatators and reamers (outer diameters: from 3 to 7 mm). The instrument position was kept under radiographic control while continuously switching from lateral to AP views **(**Fig. 2,3**)**. The reamers were used to cross the pedicle and penetrate the full width of the bone. The breaking of the medial pedicle wall, and thus the access to the intracanal space, was confirmed by simultaneously feeling the loss of resistance by hand and real-time fluoroscopic confirmation.

**Fig. 4:**
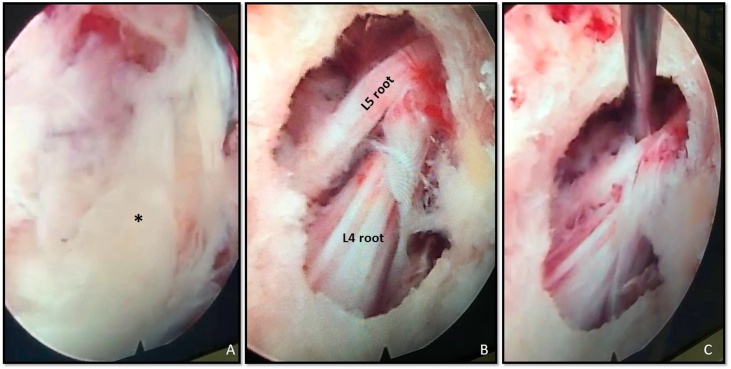
**A.** medial pedicle wall access to the spinal canal. Endoscopic first look: visible the herniated fragment (*: herniated disc fragment). **B**.nerve roots floating freely in the spinal canal after herniated fragment retrieval. **C**. spinal canal inspection with a probe for additional fragments search.

We then inserted the endoscope working cannula (8.0 mm in outer diameter) and the camera (Fig. 2,4). The herniated fragment was clearly visible (Fig. 4**, A**). We mobilized the disc fragment with the probe and the root retractor and then removed it with small forceps. The decompression was considered successful when we saw the nerve root pulsating freely in the epidural space **(**Fig. 4**, B),** after having checked the intraspinal space for additional fragments **(**Fig. 4**, C**). We closed with a resorbable suture.

Late postoperative MRI showed satisfactory decompression **(**Fig. 1**, C)** and no sign of additional instability or fractures. Patient radiculopathy entirely resolved after the surgery – postoperative pain was negligible after the first 6 h and 24 h and at the 8-month follow-up.

## 3 Discussion

Endoscopic spinal surgery for lumbar spondylosis has gained popularity [6,7], even if some surgeons still believe it should be reserved for select cases [1,8]. Technological developments have made possible to remove disc herniations distal to the intervertebral space, overcoming anatomical barriers, as is the case with down-migrated or up-migrated lumbar herniations. These disc fragments are sequestrated medial to the pedicle and may be missed with a transforaminal approach [9]. In those cases, surgeons must access the spinal canal by removing the superior portion of the inferior pedicle or by performing controlateral transforaminal access or an interlaminar fenestration. FETD, by crossing the medial wall of the pedicle [[9], [10], [11]], allows direct access into the canal at all levels of the lumbar spine, including L1-L2 and L5-S1 [10].

To date, only 26 patients have been described [9,11,12]. In seven cases, the herniation was localized at L3-L4 level (one L3-L4 juxtafacet cyst), 15 at L4-L5, and four at L5-S1. Age at intervention ranged from 31 to 78 years, with almost all herniated fragments migrated caudally (80 %). The vast majority of patients had a satisfactory outcome (∼ 90 %). Only two patients (one L3-L4 caudal fragment and an L4-L5 caudal fragment) underwent revision surgery through a transforaminal approach for recurrent radiculopathy. None had surgery-related complications. Over follow-up (2–12 months), no pedicle fractures were documented. The mean operative time was < 50 min (8), and the patient position (lateral vs. prone) was based on surgeon preference.

FETD was initially performed in lateral patient decubitus with the spinal needle positioned dorsally to the transverse process to reach the pedicle directly. Only in those cases with mild to severe lumbar scoliosis and vertebra rotation, or when the S1 pedicle has to be reached, it was preferable to enter the pedicle anterior to the transverse process. However, the prone decubitus is more comfortable, and the related three-dimensional orientation is similar to that of the conventional percutaneous transpedicular screw placement, which is more familiar for surgeons. [12].

Local anesthesia and conscious sedation are the best sedation options because they allow for pain and anxiety management while communicating with the patient, checking leg movements, and detecting sudden pain complaints. That is paramount because of the risk of hitting the exiting root when entering the spinal canal by drilling the medial wall of the pedicle. Also, the infiltration of the pedicle periosteum with 2% lidocaine may reduce the number of systemic drugs and improve perioperative pain management, but only if done by an expert endoscopic surgeon because of the puncture risk to the dura mater.

In FETD, perfect needle placement is mandatory for success. The trajectory is tangential to the medial wall of the pedicle, and the opening into the intra-canal surface is commonly made with an endoscopic drill rather than trephines or reamers [9]. However, as shown, reamers can be safely used to access the spinal canal.

Although open surgical approaches to disk extrusion carry the risk of destabilizing the spine due to facet removal, the transpedicular endoscopic approach gives the same chance of destabilizing the spine, by virtually exposing the patient to a pedicle fracture. However, due to the relatively low risk documented, we believe that imaging control for bone integrity or spinal contention is not required after the procedure. The only relative contraindication is a diameter of the pedicle lower than 8 mm, as this may increase fracture risk. Finally, when the endoscope is inside the bone tunnel, even mild movements of the system are highly discouraged.

Concerning indications, FETD was at first used to treat high-grade down-migrated disc herniations located medial to the pedicle and in the shoulder of the traversing nerve root [12]. The herniations are indeed easily removed when soft, in a single fragment high grade down-migrated, and near the medial pedicle wall [9]. Fragments located axillary to the traversing nerve root were instead considered an absolute contraindication [8] till recently when some authors successfully treated axillary highly up-migrated disc herniations [11]. Other relative contraindications are hypoplastic pedicle, severe canal stenosis, neurological deficits, calcified disc fragments, high iliac crest (for L5-S1 access), and severe osteoporosis.

Some authors described an alternative approach to treat down migrated lumbar disc prolapse. Kim and coworkers developed a suprapedicular circumferential opening technique for transforaminal endoscopic discectomy in which the articular process, upper pedicle and the upper-posterior margin of the lower vertebra are drilled to increase the width of the foramen and expose the ventral epidural space [13]. They achieved good to excellent clinical results for high grade inferiorly migrated lumbar disc herniations, but they stated that epidural bleeding might occur as a result of vascular engorgement.

Finally, endoscopic discectomy should not be considered a minor spinal procedure, requiring depth knowledge of spinal anatomy and endoscopic procedures, open surgery, and minimally-invasive techniques. Complications may arise during endoscopic procedures, and the surgeon should be able to modify the approach better to serve the patient in case of adverse events. Further extensive prospective studies are warranted to validate the efficacy and safety of FETD in the management of other spinal pathologies, such as drainage of epidural abscess, epidural hematoma, or when intra-canal biopsies are needed [9].

## 4 Conclusions

FETD showed excellent results in our patient and in the cases reported in the literature and could be effective in treating up-migrated and down-migrated disc herniation, as well as juxtafacet cysts, showing high feasibility and safety from any level from L1 to S1.

## Declaration of Competing Interest

No potential conflicts of interest to be declared.

## Sources of funding

No funding source.

## Ethical approval

The study is exempt from ethical approval.

## Consent

Written informed consent was obtained from the patient for publication of this case report and accompanying images.

## Author contribution

(I) Conception and design: Giordan E, Del Verme J, Billeci D; (II) Administrative support: Giordan E; (III) Provision of study materials or patients: Giordan E, Canova G; (IV) Collection and assembly of data: Giordan E, Del Verme J, Billeci D; (V) Data analysis and interpretation: Giordan E, Del Verme J, Billeci D; (VI) Manuscript writing: Giordan E, Del Verme J, Billeci D, Coluzzi F; (VII) Final approval of manuscript: All authors

## Registration of research studies

Not necessary in this case. This is a case report

## Guarantor

Dr. Enrico GIORDAN

## Provenance and peer review

Not commissioned, externally peer-reviewed

## References

[1] Rasouli M.R., Rahimi-Movaghar V., Shokraneh F., Moradi-Lakeh M., Chou R. (2014). Minimally invasive discectomy versus microdiscectomy/open discectomy for symptomatic lumbar disc herniation. Cochrane Database Syst. Rev..

[2] Xie T.-H., Zeng J.-C., Li Z.-H., Wang L., Nie H.-F., Jiang H.-S. (2017). Complications of Lumbar Disc Herniation Following Full-endoscopic Interlaminar Lumbar Discectomy: A Large, Single-Center, Retrospective Study. Pain Physician.

[3] Garg B., Nagraja U.B., Jayaswal A. (2011). Microendoscopic versus open discectomy for lumbar disc herniation: a prospective randomised study. J. Orthop. Surg. [Internet].

[4] Agha R.A., Borrelli M.R., Farwana R., Koshy K., Fowler A., Orgill D.P., For the SCARE Group (2018). The SCARE 2018 statement: updating consensus surgical CAse REport (SCARE) guidelines. Int. J. Surg..

[5] Sheahan C.G., Mathews D.M. (2014). Monitoring and delivery of sedation. Br. J. Anaesth. [Internet].

[6] Middleton S.D., Wagner R., Gibson J.N.A. (2017). Multi-level spine endoscopy. EFORT Open Rev..

[7] Patil A., Chugh A., Gotecha S., Kotecha M., Punia P., Ashok A. (2018). Microendoscopic discectomy for lumbar disc herniations. J. Craniovertebral Junction Spine.

[8] Kim M., Lee S., Kim H.-S., Park S., Shim S.-Y., Lim D.-J. (2018). A comparison of percutaneous endoscopic lumbar discectomy and open lumbar microdiscectomy for lumbar disc herniation in the korean: a meta-analysis. Biomed Res. Int..

[9] Uniyal P., Choi G., Khedkkar B. (2016). Percutaneous transpedicular lumbar endoscopy: a case report. Int. J. Spine Surg..

[10] Krzok G., Telfeian A.E., Wagner R., Iprenburg M. (2016). Transpedicular lumbar endoscopic surgery for highly migrated disk extrusions: preliminary series and surgical technique. World Neurosurg [Internet].

[11] Krzok G. (2019). Transpedicular endoscopic surgery for highly downmigrated L5-S1 disc herniation. Case Rep. Med..

[12] Quillo-Olvera J., Akbary K., Kim J.S. (2018). Percutaneous endoscopic transpedicular approach for high-grade down-migrated lumbar disc herniations. Acta. Neurochir. (Wien)..

[13] Kim H.S., Yudoyono F., Paudel B., Kim K.J., Jang J.S., Choi J.H. (2018). Suprapedicular circumferential opening technique of percutaneous endoscopic transforaminal lumbar discectomy for high grade inferiorly migrated lumbar disc herniation. Biomed Res. Int..

